# High-throughput phenotyping (HTP) identifies seedling root traits linked to variation in seed yield and nutrient capture in field-grown oilseed rape (*Brassica napus* L.)

**DOI:** 10.1093/aob/mcw046

**Published:** 2016-04-06

**Authors:** C. L. Thomas, N. S. Graham, R. Hayden, M. C. Meacham, K. Neugebauer, M. Nightingale, L. X. Dupuy, J. P. Hammond, P. J. White, M. R. Broadley

**Affiliations:** ^1^Plant and Crop Sciences, School of Biosciences, University of Nottingham, Sutton Bonington Campus, Loughborough LE12 5RD, UK,; ^2^Ecological Sciences, The James Hutton Institute, Invergowrie, Dundee DD2 5DA, UK,; ^3^Elsoms Seeds Ltd, Pinchbeck Rd, Spalding PE11 1QG, UK,; ^4^School of Agriculture, Policy and Development, University of Reading, Whiteknights, PO Box 237, Reading RG6 6AR, UK and; ^5^Distinguished Scientist Fellowship Program, King Saud University, Riyadh 11451, Kingdom of Saudi Arabia

**Keywords:** *Brassica napus* (OSR, canola), lateral root density, mineral concentration, primary root length, seed yield

## Abstract

**Background and Aims** Root traits can be selected for crop improvement. Techniques such as soil excavations can be used to screen root traits in the field, but are limited to genotypes that are well-adapted to field conditions. The aim of this study was to compare a low-cost, high-throughput root phenotyping (HTP) technique in a controlled environment with field performance, using oilseed rape (OSR; *Brassica napus*) varieties.

**Methods** Primary root length (PRL), lateral root length and lateral root density (LRD) were measured on 14-d-old seedlings of elite OSR varieties (*n* = 32) using a ‘pouch and wick’ HTP system (∼40 replicates). Six field experiments were conducted using the same varieties at two UK sites each year for 3 years. Plants were excavated at the 6- to 8-leaf stage for general vigour assessments of roots and shoots in all six experiments, and final seed yield was determined. Leaves were sampled for mineral composition from one of the field experiments.

**Key Results** Seedling PRL in the HTP system correlated with seed yield in four out of six (*r* = 0·50, 0·50, 0·33, 0·49; *P* < 0·05) and with emergence in three out of five (*r* = 0·59, 0·22, 0·49; *P* < 0·05) field experiments. Seedling LRD correlated positively with leaf concentrations of some minerals, e.g. calcium (*r* = 0·46; *P* < 0·01) and zinc (*r* = 0·58; *P* < 0·001), but did not correlate with emergence, general early vigour or yield in the field.

**Conclusions** Associations between PRL and field performance are generally related to early vigour. These root traits might therefore be of limited additional selection value, given that vigour can be measured easily on shoots/canopies. In contrast, LRD cannot be assessed easily in the field and, if LRD can improve nutrient uptake, then it may be possible to use HTP systems to screen this trait in both elite and more genetically diverse, non-field-adapted OSR.

## INTRODUCTION

Direct phenotypic selection for yield, appearance and quality is routinely conducted on the roots of domesticated crops such as carrot (*Daucus carota*; Stein and Nothnagel, 1995) and cassava (*Manihot esculenta*; Nassar and Ortiz, 2007). For most non-root crops, including cereals and legumes, direct phenotypic selection of root traits has not been widely attempted. However, indirect selection of root traits has undoubtedly supported historical yield increases (Lynch, 2007, 2011). Examples of root traits shown to correlate with improved field performance include thicker, longer roots in rice (*Oryza sativa*; Steele *et al.*, 2013), increased root hair elongation in maize (*Zea mays*; Hochholdinger *et al*., 2008) and shallower roots under low phosphorus (P) conditions in soybean (*Glycine max*; Zhao *et al*., 2004) and common bean (*Phaseolus vulgaris*; Bonser *et al*., 1996; Ho *et al*., 2005; Miguel *et al*., 2013).

In rice, beneficial root traits have recently been introgressed successfully into elite crop varieties. For example, multiple quantitative trait loci (QTL) associated with thicker and longer roots were introgressed from a *japonica* into an *indica* line via marker-assisted selection (Steele *et al*., 2007, 2013). This led to improved field performance under favourable field conditions and a new rice variety (Birsa Vikas Dhan 111) has subsequently been released. The *Phosphorus Uptake 1* (*PUP1*) QTL has been introgressed into commercial rice varieties, increasing root growth and yield in P-deficient soils (Chin *et al*., 2010). The *Deeper Rooting 1* (*DRO1*) QTL has been back-crossed into a shallow-rooting rice variety, increasing yield under drought conditions by increasing root depth (Uga *et al*., 2013; Arai-Sanoh *et al*., 2014). There are fewer reports on the introgression of root traits in other crops; however, QTL-linked root traits in wheat (*Triticum aestivum*; Placido *et al*., 2013) and chickpea (*Cicer arietinum*; Varshney *et al*., 2013) have been introduced into elite varieties and shown to improve drought tolerance.

Direct selection of root traits under field conditions is possible, albeit challenging in many environments. For example, low-cost root excavations can be made using hand-held tools (‘shovelomics’; Trachsel *et al*., 2011; Wishart *et al*., 2013; Bucksch *et al*., 2014). More involved methods of observing root traits include soil-coring/washing/breaking (Gregory and Eastham, 1996; Ostonen *et al*., 2007; Watt *et al*., 2013; Wasson *et al*., 2014) and the use of interfaces such as ‘windows’, trenches and minirhizotrons (Jose *et al*., 2001; Dupuy *et al*., 2010; Maeght *et al*., 2013; Vansteenkiste *et al*., 2014). *In situ* proxy measurements for studying root traits include: (1) using buried herbicides to monitor root depth (Maeght *et al*., 2013; Al-Shugeairy *et al*., 2014); (2) electromagnetic induction to estimate root biomass from water depletion (Srayeddin and Doussan, 2009; Shanahan *et al*., 2015); (3) measuring root capacitance (Dietrich *et al*., 2012, 2013); and (4) quantifying plant root DNA concentrations (Huang *et al*., 2013). *Ex situ* methods for studying the roots of soil-grown plants include X-ray microcomputed tomography (µCT; Tracy *et al*., 2010; Mooney *et al*., 2012) and magnetic resonance imaging (MRI; Rogers and Bottomley, 1987; Metzner *et al*., 2015), which may have potential for field-grown crops in the future. Despite these methodological advances, measuring root traits in the field is still a major constraint to direct phenotypic selection for crop improvement due to cost, time and logistical implications (e.g. soil hardness).

A further constraint to selecting root traits directly is the paucity of phenotypic diversity in many crops arising from domestication/genetic bottlenecks (Bus *et al*., 2011). However, more diverse genetic material cannot easily be studied in the field when it is not adapted to local environmental conditions. The use of high-throughput phenotyping (HTP) systems *in vitro* can potentially overcome this constraint (Hammond *et al*., 2009; Chen *et al*., 2011; Koscielny and Gulden, 2012; Downie *et al*., 2015). Numerous HTP techniques have been developed, including rhizotrons for soil-grown plants (Nagel *et al*., 2012; Lobet and Draye, 2013) and transparent media, which enable 3D root imaging *in vitro* (Iyer-Pascuzzi *et al*., 2010; Clark *et al*., 2011; Downie *et al*., 2012). Simpler, 2D HTP systems include agar plate-based systems (Armengaud *et al*., 2009; French *et al*., 2009; Shi *et al*., 2013) and ‘pouch and wick’ systems, in which roots are grown on filter paper, suspended in nutrient solution, which is surrounded by a pouch that conceals roots from light (Bonser *et al*., 1996; Hund *et al*., 2009; Miguel *et al*., 2013; Watt *et al*., 2013; Adu *et al*., 2014; Le Marie *et al*., 2014; Atkinson *et al*., 2015). An important observation from these HTP studies is that root traits are often highly variable, and so require large numbers of replicates to assess genotypic versus non-genotypic sources of variation (Adu *et al*., 2014). Whilst such HTP systems cannot allow the assessment of roots in soil, they can enable promising germplasm to be identified which could then be crossed into field-adapted material for direct testing in the field. However, few HTP studies have been designed to test the associations between root traits and field performance in elite germplasm explicitly (White *et al*., 2013*b*).

The aim of this study was to test a low-cost HTP technique for screening seedling root traits in oilseed rape (OSR; *Brassica napus*). *Brassica napus* is a complex allotetraploid hybrid of its diploid progenitor species, *Brassica rapa* and *Brassica oleracea* (Chalhoub *et al*., 2014). We selected OSR for study as it is an important crop, providing 18 % of global vegetable oil for human consumption, plus industrial oils, biodiesel, lubricants and animal feeds. Over 60 million tonnes of seed are produced annually worldwide (FAOSTAT, 2015). In the UK, it is grown on >700 000 ha, primarily in rotation with wheat. Yields of OSR in the UK have remained constant since 1990, at ∼3·2–3·4 t ha^−1^, and it is thought there is scope for improvement based on improving water and nitrogen (N) acquisition, especially post-anthesis, through improved rooting traits (Berry *et al*., 2010; White *et al.*, 2013*b*). There is limited published information on OSR seedling root traits in relation to yield, although Koscielny and Gulden (2012) reported a relationship among eight varieties of *B. napus* between root length measured on 7-d-old seedlings and their seed yield in the field in Canada. Previous HTP studies on *Brassica oleracea*, *B. napus and B. rapa* show that QTL for seedling root traits correlate with plant shoot biomass (Hammond *et al*., 2009; Shi *et al*., 2013; Adu *et al*., 2014). The work presented here extends these observations to test whether root architectural traits measured on seedlings of *B. napus* relate to key crop characteristics in the field.

## MATERIALS AND METHODS

### Plant material

Thirty-two elite winter OSR varieties were grown in an HTP pouch and wick system in a controlled environment and at two UK field sites each year, for three years. Root traits measured in the HTP system included primary root length (PRL), lateral root length (LRL) and lateral root density (LRD). Traits measured in the field experiments included final seed yield in all experiments and assessments of early crop establishment and nutrient acquisition in a subset of experiments. The OSR varieties were chosen primarily based on varieties in the Recommended List (RL) of the UK Agriculture and Horticulture Development Board (AHDB), to enable root traits measured in the HTP system to be tested in the field on UK-adapted material. Varieties included conventional types (*n* = 20), recombinant hybrid types (*n* = 11) and fodder types (*n* = 1).

### HTP experiments

**Fig. 1.**
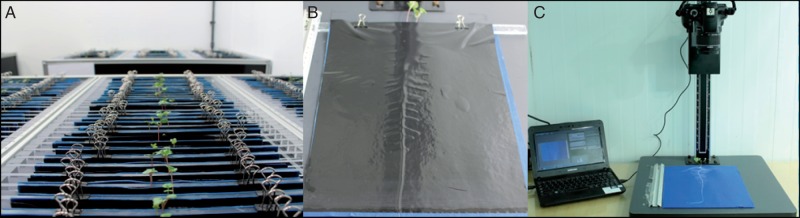
(A) *Brassica napus* seedlings growing in the hydroponic pouch and wick system. (B) Growth pouch 14 d after sowing (DAS). (C) Stand-mounted camera at fixed height above germination paper with root 14 DAS.

A pouch and wick hydroponic-based HTP system (Atkinson *et al*., 2015) was deployed in this study (Fig. 1). This system comprised growth pouches assembled from blue germination paper (SD7640; Anchor Paper Company, St Paul, MN, USA), re-cut to 24 × 30 cm and overlaid with black polythene (Cransford Polythene Ltd, Woodbridge, UK). Along their shorter edges, the paper and polythene were clipped together to each side of an acrylic bar (Acrylic Online, Hull, UK) using bulldog-type fold-back clips. The growth pouches were suspended above plastic drip trays, supported within lightweight aluminium/polycarbonate frames, as described in Atkinson *et al*. (2015). Each drip tray contained 2 L of 25 % strength Hoagland’s solution (Hoagland’s No. 2 Basal Salt Mixture, Sigma Aldrich, Dorset, UK) made with deionized water. Drip trays were replenished with 500 mL of deionized water every 3 d. Prior to sowing, the pouches were suspended above the nutrient solution for a minimum of 4 h to become fully saturated. A single seed was sown in the middle of the upper edge of each germination paper by pressing the seed into the paper. Within each aluminium frame, nine drip trays were used, arranged in three columns and three rows. Pouches were allocated randomly to drip trays, 10 or 11 pouches per drip tray, thus giving 96 pouches and 192 plants per frame. A total of four frames were used in each experimental run, giving a potential sample size of 768 plants per run within a single controlled-environment room. The controlled-environment room was 2·2 m wide, 3·3 m long and 3·0 m high, set to a 12-h photoperiod with 18/15 °C day/night temperatures and relative humidity of 60–80 %. Photosynthetically active radiation (PAR; measured at plant height with a 190 SB quantum sensor; LI-COR Inc., Lincoln, NE, USA) was 207 µmol m^−2 ^s^−1^, generated by 400-W white fluorescent lamps (HIT 400w/u/Euro/4K, Venture Lighting, Rickmansworth, UK). Fourteen days after sowing (DAS), the polythene sheets were removed from all pouches and images were taken of the germination paper and root system for downstream image analysis. Images were taken using a digital single-lens reflex (DSLR) camera (Canon EOS 1100D, Canon Inc., Tokyo, Japan) with a focal length of 35 mm at a fixed height of 75 cm, using Canon software.

### Root image processing

The root images from the HTP system were renamed with each sample’s unique experimental design information using Bulk Rename Utility (Version 2.7.1.3, TGRMN Software, www.bulkrenameutility.co.uk). Images were cropped by reducing extraneous pixels on bulked images, using XnConvert (Version 1.66, www.xnconvert.com). Cropped images were analysed using RootReader2D (RR2D; Clark *et al.*, 2013). First, a batch process was carried out which automatically ‘thresholds’, ‘skeletonizes’ and ‘builds segments’ of all images in bulk. The root system was then measured on individual images by placing a marker at the base and tip of the primary root. From these markers, RR2D automatically calculates PRL, LRL of all laterals, and lateral root number (LRN). Further traits calculated from these data included total root length (TRL = PRL + LRL), mean lateral root length (MLRL = LRL/LRN) and LRD (LRN/PRL). A database was developed which integrated the experimental design information from the image name with the RR2D measurements for each sample, using a programming script (2.7.10; Python Software Foundation, www.python.org).

### Field experiment locations

Experiments were conducted in 2012–13, 2013–14 and 2014–15 at two field sites each year: Bingham (Nottinghamshire) and Deeping St Nicholas (Lincolnshire) in 2012–13 and 2013–14; the Bingham site was replaced by a site at Harlaxton (Lincolnshire) in 2014–15 (Table 1). Experiments were managed by Elsoms Seeds Ltd (Spalding, UK) according to standard National List protocols for winter oilseed rape. Untreated seeds of each variety were sown at 60 seeds m^−2^ in plots of 12 m length × 1·85 m width, allocated to a randomized-block design of three replicates. Standard field assessments included final seed yields for all experiments, and emergence (scored 5 weeks after drilling by an experienced oilseed breeder on a subjective scale of 1–8, where 8 represents best possible emergence) for most experiments. Additional trait assessments are described in the following sections.

**Table 1.** Locations, timings, soil types, soil pH and cation exchange capacity (CEC) of sites used for the field experiments conducted in 2012–13, 2013–14 and 2014–15

Site/grid reference	Sowing date	Harvest date	Soil type^a^	pH	CEC(meq 100 g^−1^)
Bingham SK 6 955 738 274	30/8/2012 30/8/2013	17/8/2013 17/8/2014	Slightly acid, loamy and clayey soils with impeded drainage. Fertility: moderate to high	7·0	14·9
Deeping TF 2 170 017 100	6/9/2012 6/9/2013 3/9/2014	29/8/2013 29/8/2014 28/7/2015	Loamy and clayey soils of coastal flats with naturally high groundwater. Fertility: lime-rich to moderate	7·6	32·1
Harlaxton SK 8 891 134 066	27/8/2014	3/8/2015	Slowly permeable seasonally wet slightly acid but base-rich loamy and clayey soils. Fertility: moderate	7·2	13·1

^a^Data from Landis: http://www.landis.org.uk/soilscapes/#.

### General early vigour assessments of shoots and roots in the field

Plants were sampled at an early growth stage (∼6- to 8-leaf stage) from every plot, before roots became too large to excavate easily, with six to eight plants sampled per plot. From the mid-sections of each plot, roots and shoots were removed using a garden fork and stored in plastic bags at <5 °C. Subsequently, roots were washed with detergent (1 % v/v; Teepol, Kent, UK). For the 2012–13 and 2013–14 experiments, roots were photographed as described for the HTP experiments using the DSLR camera. Images of field-sampled roots were analysed using ImageJ (Rasband, 1997–2014, http://rsbweb.nih.gov/ij/docs/faqs.html). The length of the longest root axis (i.e. PRL) and the base diameter of the primary root (PRBD), were traced using the ‘segmented line’ tool. The mean diameter of the primary root (PRMD) was calculated by dividing the root length into quarters and obtaining the mean root diameter at these points. Dry weights of roots and shoots were obtained for all field experiments; samples were oven-dried at 50 °C for ∼48 h and weighed.

### Leaf sampling in the field and preparation for elemental analysis

Leaves were sampled at flowering, prior to seed-set (June 2014) from every plot at the Bingham site from the 2013–14 experiment. For each of the 96 plots, a composite sample was taken comprising one fully expanded leaf removed from the tops of the stems of four randomly selected plants. Leaf samples were dried in paper bags at 50 °C for 48 h and hand-crushed to a powder within the bag, and 0·1 g was subsampled for elemental analysis. Samples were digested using a solution of 1 mL of 30 % H_2_O_2_, 2 mL of 50 % trace analysis grade HNO_3_ and 1 mL of Milli-Q water (18·2 MΩ·cm; Fisher Scientific UK Ltd, Loughborough, UK). Solutions were placed in a Multiwave 3000 microwave with a 48-vessel 48MF50 rotor (Anton Paar GmbH, Graz, Austria) and heated for 45 min at a controlled pressure of 2 MPa, within vessels comprising perfluoroalkoxy liner material and polyethylethylketone pressure jackets (Anton Paar GmbH). Digested samples were diluted to 15 mL with Milli-Q water and stored at room temperature. Immediately prior to analysis, digested samples were diluted 1-in-10 with Milli-Q water. The concentrations of 28 elements were obtained using inductively coupled plasma mass spectrometry (ICP-MS; Thermo Fisher Scientific iCAPQ, Thermo Fisher Scientific, Bremen, Germany): Ag, Al, As, B, Ba, Ca, Cd, Cr, Co, Cs, Cu, Fe, K, Mg, Mn, Mo, Na, Ni, P, Pb, Rb, S, Se, Sr, Ti, U, V and Zn. For each data point, element-specific operational blank concentrations (ICP-run means) were subtracted. An external Certified Reference Material was included (IAEA-359 Cabbage; LGC, Teddington, UK).

### Statistical analyses

Pearson’s correlation coefficients were calculated for root traits of plants grown in the HTP system and emergence, root and shoot traits, and yield in field experiments. One variety (ID 29) was excluded from data analysis because it is a forage type with low seed yield. Variance components were calculated for all HTP traits using a residual maximum likelihood (REML) procedure, with all factors classed as random factors so that the proportional contribution of genotype to overall variation in traits could be determined. The experimental power to compare root traits of different genotypes was determined for both the HTP system and the field. For this, a *t*-test sample-size calculation was used, whereby the measure of variance was the residual mean square from the REML procedure and the response to be detected was set as a percentage of the grand mean of all varieties; data were plotted as contour plots. All statistical analyses were conducted using GenStat 15th Edition (VSN International Ltd, Hemel Hempstead, UK).

## RESULTS

A total of 1200 images were obtained from the HTP system and 2428 images from the field experiments. In the HTP system, recombinant hybrid varieties had a longer PRL than conventional varieties (*t*-test, *F* = 2·07; *P* < 0·001; Table 2) and had greater emergence (*t*-test, *F* = 1·63; *P* < 0·05; Supplementary Data Table S1) and seed yield (*t*-test, *F* = 1·87; *P* < 0·05; Table 2) under field conditions.

**Table 2.** *Brassica napus* varieties grown in the HTP system; variety type; recommended area for cultivation in the UK; year of first inclusion the recommended list (RL), primary root length (PRL; mean ± s.d.); and final seed yield in the field. HTP data are from a pouch and wick system with seedlings imaged at 14 DAS (*n* = ∼40). Field data are means of six field experiments (mean ± s.d.). Varieties are ranked by PRL

Variety	Variety type	Year on RL	Recommended area	PRL (cm)	Yield (t ha^−1^)
16	Recombinant hybrid	2013–14	Club root-infected land	18·5 ± 6·7	5·3 ± 0·9
8	Recombinant hybrid	2011–12	All UK	17·4 ± 6·0	5·5 ± 0·8
18	Recombinant hybrid	2004–05	Club root-infected land	17·3 ± 8·2	5·2 ± 1·8
29	Fodder	Not on RL	–	17·3 ± 5·7	–
11	Conventional	2009–10	E/W	17·2 ± 6·7	5·3 ± 0·8
1	Recombinant hybrid	2013–14	E/W	17·2 ± 6·4	4·9 ± 0·5
9	Recombinant hybrid	2012–13	E/W	17·2 ± 5·3	6·0 ± 0·6
17	Conventional	2005–06	All UK	16·6 ± 5·3	4·6 ± 0·5
25	Recombinant hybrid	2009–10	N	16·3 ± 6·7	5·2 ± 0·7
14	Conventional	2005–06	All UK	16·2 ± 7·0	5·3 ± 0·3
10	Conventional	2010/11	E/W	16·1 ± 5·2	5·1 ± 1·0
2	Conventional	Not on RL	–	15·9 ± 5·3	5·2 ± 0·7
12	Recombinant hybrid (semi-dwarf)	2012/13	E/W	15·6 ± 8·3	4·9 ± 0·7
15	Conventional	2005/06	All UK	15·5 ± 5·0	4·9 ± 0·6
24	Conventional	Not on RL	–	15·4 ± 4·7	5.1 ± 0·8
21	Recombinant hybrid	2013/14	E/W	15·3 ± 6·6	5·5 ± 0·5
13	Recombinant hybrid	2008/09	All UK	15·2 ± 5·3	5·7 ± 0·7
5	Conventional	2011/12	E/W	14·9 ± 6·5	5·2 ± 0·5
3	Conventional	Not reported	E/W	14·8 ± 4·8	5·4 ± 0·5
26	Conventional	2008/09	N	14·7 ± 5·7	5·1 ± 0·7
4	Conventional	2012/13	E/W	14·7 ± 5·8	5·1 ± 0·9
28	Conventional	Not reported	–	14·1 ± 6·1	5·1 ± 0·7
27	Conventional	Not reported	E/W	14·1 ± 3·8	5·1 ± 0·7
6	Conventional	2009/10	E/W	14·1 ± 4·4	5·1 ± 0·4
32	Conventional	2004/05	All UK	13·7 ± 3·9	4·6 ± 0·8
23	Conventional	Not on RL	–	13·2 ± 6·5	4·7 ± 1·0
19	Conventional	Not on RL	–	12·9 ± 5·0	4·3 ± 0·8
20	Conventional	2010/11	N	12·5 ± 5·1	4·9 ± 1·1
7	Conventional	2010/11	E/W	12·4 ± 5·7	5·2 ± 0·8
30	Conventional	2005/06	All UK	12·3 ± 4·7	4·6 ± 0·5
31	Conventional	Not on RL	–	11·0 ± 4·0	4·9 ± 0·5
22	Recombinant hybrid	2011/12	E/W	–	5·5 ± 0·5

–, not applicable.

N, North Region, comprising Scotland, Northern Ireland and Northern England; E/W, East/West Region, comprising England (south of Teesside) and Wales.

Variety 18 did not grow in the field in 2012–13 (i.e. *n* = 4). Variety 22 did not grow in the HTP system.

**Fig. 2.**
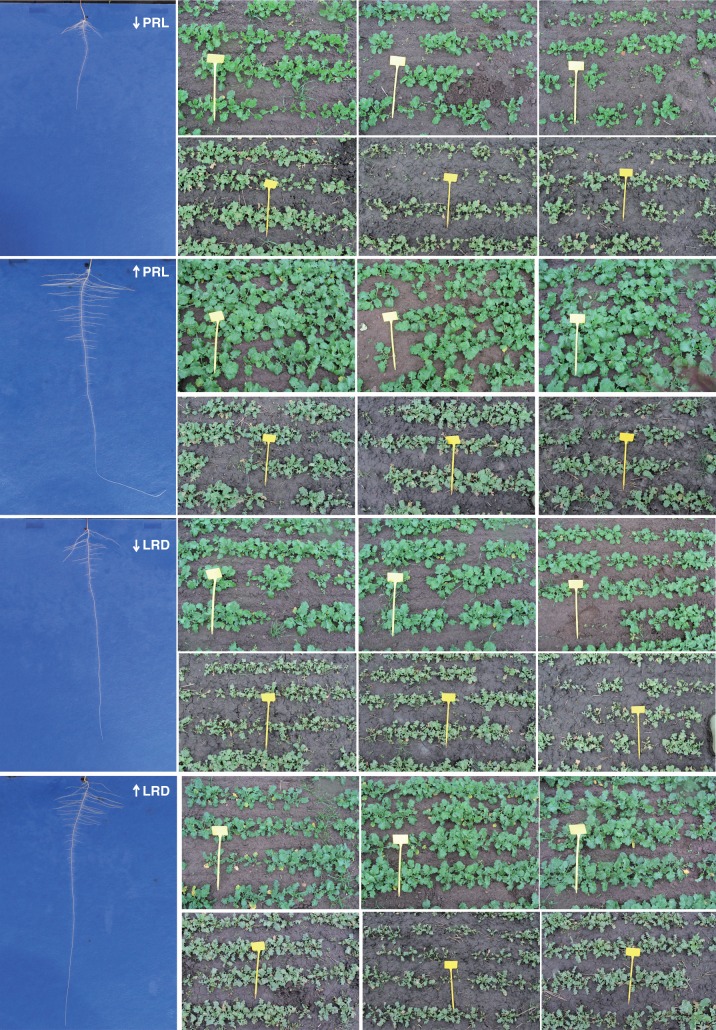
Varieties ID 31 and ID 16 with the minimum (↓PRL) and maximum (↑PRL) primary root length and varieties ID 15 and ID 17 with the minimum (↓LRD) and maximum (↑LRD) lateral root density, when grown for 14 d in the pouch and wick system (illustrative examples; vertical axis = 32 cm). Field images are triplicate plots of the same varieties at two UK field sites (2012–13): Nottinghamshire (greener, more established plots, upper panels); Lincolnshire (paler, less-established plots, lower panels; markers = 20 cm).

**Fig. 3.**
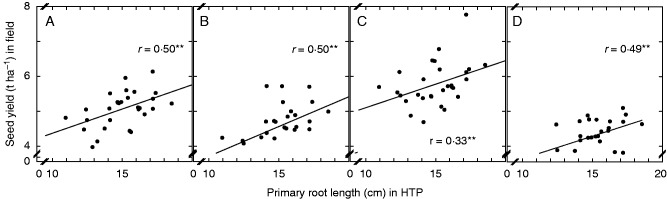
Seed yield of *Brassica napus* varieties grown in the field as a function of primary root length in the HTP system. Field data are means of triplicate plots at Bingham and Deeping in 2012–13 (A, B), at Bingham in 2013–14 (C) and at Deeping in 2014–15 (D). HTP data are means of ∼40 replicates imaged 14 d after sowing. **P < 0·01.

There were general overall positive relationships between root traits measured in the HTP system and field traits for most sites and years. For example, there were positive relationships between PRL and emergence in three of the field experiments (*r* = 0·59, *r* = 0·22, *r* = 0·49; Supplementary Data Table S1). Thus, whilst plants established much more rapidly at Bingham than at Deeping in 2012–13, varieties with the longest PRL showed better emergence and crop establishment at both sites (Fig. 2). There were also significant positive relationships between PRL and final seed yield in four of the field experiments (*r* = 0·50, *r* = 0·50, *r* = 0·33, *r* = 0·49) (Fig. 3; Supplementary Data Table S1).

The relationships among root traits measured within and between the HTP and all field experiments are shown in Table 3. Within the HTP system, there were significant positive relationships between most root length-related traits. For example, there were positive relationships between PRL and TRL (*r* = 0·84). However, there was no significant correlation between PRL and LRD. The only significant negative correlation among the root traits measured in the HTP system was between LRD and MLRL (*r* = −0·38), which suggests a potential trade-off for these traits. Consistent with these observations, LRD measured in the HTP system did not correlate with yield or any other traits measured in the field, such as emergence or root and shoot dry weights at the 6- to 8-leaf stage. Field experiments indicated that early growth correlated positively with final seed yields. Thus, root and shoot dry weights were correlated at the 6- to 8-leaf stage (*r* = 0·90), and both these traits were correlated with final seed yield (*r* = 0·49, *r* = 0·46, respectively).

**Table 3.** Pearson correlation coefficients (*r*) between root traits of *Brassica napus* varieties grown in the HTP system, and root and shoot traits of varieties grown in all six field experiments.

		PRL	LRD	MLRL	TRL						
HTP	PRL	–									
	LRD	0·20	–								
	MLRL	0·14	–0·38*	–							
	TRL	0·84***	0·45**	0·32	–						
Field	PRL	0·32	0·31	−0·22	0·26	–					
	PRBD	0·42*	0·08	0·06	0·33	0·68***	–				
	RDW	0·51***	0·02	0·01	0·42**	0·34	0·58***	–			
	SDW	0·49***	−0·07	0·08	0·37**	0·09	0·41*	0·90***	–		
	EM	0·55***	0·16	0·12	0·50***	0·70***	0·81***	0·64***	0·50**	–	
	YIELD	0·35	−0·05	0·06	0·26	0·26	0·39*	0·49**	0·46**	0·66***	–
		PRL	LRD	MLRL	TRL	PRL	PRBD	RDW	SDW	EM	YIELD

***, ** and * represent *P* < 0·001, *P* < 0·01 and *P* < 0·05, respectively.

**Fig. 4.**
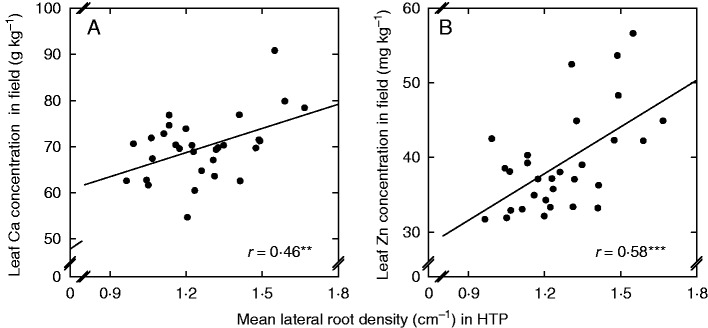
Leaf dry weight concentrations of calcium (A) and zinc (B) in the leaves of *Brassica napus* varieties grown in the field as a function of lateral root density in the HTP system. Field data are means of triplicate plots at Bingham in 2013–14. HTP data are means of ∼40 replicates imaged 14 d after sowing. **P < 0·01; ***P < 0·001.

The LRD measured in the HTP system was significantly positively related to leaf mineral concentrations, e.g. calcium (*r* = 0·46) and zinc (*r* = 0·58), measured in the field (Fig. 4; Supplementary Data Table S2). Given that no yield or emergence penalties were observed among varieties with greater LRD (Table 3), it is plausible that varieties with greater LRD were able to take up these nutrients more efficiently.

**Fig. 5.**
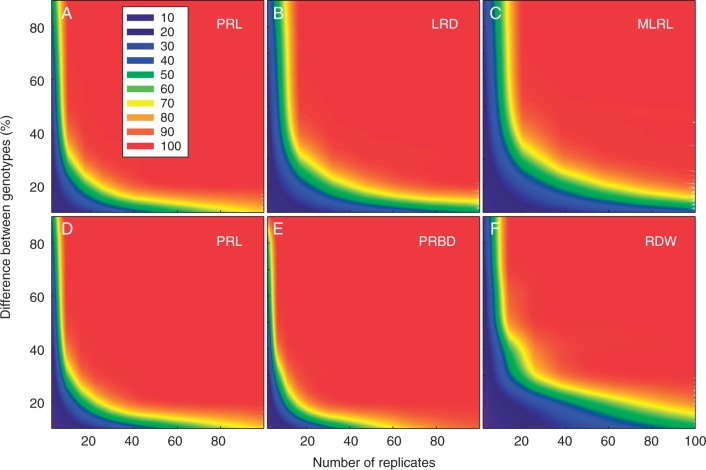
The experimental power (*z*-axis, legend inset in percentage units) to detect percentage differences in trait means between varieties (*y*-axis) as a function of the number of replicates (*x*-axis). (A, B, C) HTP system. (D, E, F) Field data from Bingham 2012–13.

The experimental power to detect differences in trait means between varieties was determined as a function of the number of replicates for the HTP system and for one of the field sites (Bingham 2012–13; Fig. 5). When measured in the HTP system, fewer replicates are needed to detect differences in PRL than in LRD and MLRL. For example, to have an 80 % power to detect 30 % differences in PRL, LRD and MLRL would require 15, 25 and 36 replicates, respectively. In the field experiments, fewer replicates are needed to detect differences in PRBD than in PRL or root dry weight (RDW). For example, to have an 80 % power to detect 30 % differences in PRBD, PRL and RDW would require 11, 14 and 34 replicates, respectively. Varietal (type + variety) factors accounted for relatively little of the total variation in root traits in the HTP system (≤9 %; Table 4 and Supplementary Data Table 3).

**Table 4.** Variance component analysis of *Brassica napus* root traits in the HTP system, and root and shoot traits in the field from all six field experiments. Variety type component represents recombinant hybrid or conventional. Environment component represent variation allocated to all experimental design factors

	HTP system				Field			
Variance component (%)	PRL	LRD	MLRL	TRL	PRL	PRBD	RDW	SDW
Variety type	6	1	0	3	0	3	2	1
Variety	3	6	1	5	2	5	1	n.f.
Year	–	–	–	–	n.f.	n.f.	22	16
Site	–	–	–	–	3	30	31	43
Environment	17	10	5	18	15	9	11	13
Residual	74	83	94	73	80	54	33	27

n.f., not fitted during residual maximum likelihood procedure.

## DISCUSSION

The estimated time for obtaining root data (sowing, imaging, data analysis) from the HTP system described in this paper was ∼2 min per individual plant. The costs of consumables and growth space were <£1 per plant. This compares to ∼20 min per plant to extract, wash, image and analyse root data when grown in the field (excluding sowing, crop management and harvesting).

Hybrid OSR types tended to have greater PRL than conventional OSR types in the HTP system (Table 2). Koscielny and Gulden (2012) also observed greater root growth in hybrids compared with open-pollinated varieties in OSR seedlings. Similarly, Hoecker *et al*. (2006) observed heterosis (hybrid vigour) in PRL and LRD of maize 4 d post-germination. Thus, some of the variation in OSR root length traits could be due to hybrid vigour; this hypothesis could be tested on larger collections of OSR germplasm. Two OSR varieties bred for resistance to club root disease were among the top three varieties ranked for PRL (Table 2), indicating that these genotypes have vigorous roots. The interactions between all soil-borne diseases and root traits is clearly an area that warrants further investigation; for example, diseases such as *Rhizoctonia solani* can cause profound damage to primary roots of OSR (Sturrock *et al.*, 2015). Variety ID 29 had one of the longest PRLs in the HTP system and also had the largest shoot dry weight (SDW) and one of the largest RDWs in most field experiments at the 6- to 8-leaf stage. However, ID 29 was not considered in subsequent analyses because it is a forage variety type and therefore much lower-yielding in terms of seed.

Length-related root traits measured in the HTP system were associated with traits related to general vigour in the field (Table 3). Thus, correlations were routinely seen between seedling root length traits and growth-related traits in the field, including emergence (2–3 weeks post-sowing), SDW and RDW at the 6- to 8-leaf stage, and final seed yield in most of the six field site × year combinations. This observation is again consistent with Koscielny and Gulden (2012), who compared four hybrid and four open-pollinated OSR varieties and found that PRL at the 1- to 2- and 3- to 4-leaf stages was a predictor of seed yield. In diploid *B. oleracea*, total root length correlated strongly with shoot biomass in pot-grown plants under both low and high external P supply (Hammond *et al*., 2009). In the variance component analysis (Table 4), variety type (i.e. hybrid or conventional) had the largest effect on PRL, which is consistent with PRL having the strongest relationship to general field vigour of all traits in the HTP system. This current study is based on a relatively small number of genotypes and further field experiments and yield stability assessments, on more variety × location combinations, are required to understand how factors, e.g. weather and disease, influence these undoubtedly complex relationships. For example, seed yield at Deeping in 2013–14 correlated negatively (albeit non-significantly) with seed yield in all of the other site × year combinations. Ultimately, the direct test of the value of HTP will be when root traits from non-field-adapted material have been crossed into elite varieties to improve field performance at multiple locations.

Roots of young plants in the field must acquire water and nutrients quickly, especially in small-seeded species with limited nutrient reserves (Wright *et al*., 1996; White and Veneklaas, 2012). So it is not surprising that seedling root length traits explain general early vigour under field conditions in OSR, which then explains some of the variation in final yield. Correlations between seedling development under glasshouse and field conditions, including final yield and quality traits, has also been reported in a large diversity panel of *B. napus* (Körber *et al*., 2012). Thus, given that establishment and early vigour traits can be measured easily on shoots/canopies, root length traits in OSR might have limited additional selection value for field-adapted germplasm. However, seedling root length can still be measured rapidly and cheaply in an HTP system alongside other traits (e.g. LRD), especially when large numbers of varieties are being compared. Furthermore, an HTP system is advantageous for studying genotypes that are not well adapted to field conditions and therefore require a homogeneous/controlled environment. However, there are, of course, substantial year and site effects that can limit the scope for detecting genotype effects even in field adapted material (Table 4).

In contrast to length-related traits, LRD measured in the HTP system was not significantly correlated with establishment, SDW or RDW at the 6- to 8-leaf stage, or seed yield in the field. However, LRD measured in the HTP system was influenced significantly by variety (Table 4). Furthermore, significant positive correlations between LRD and leaf concentrations of three mineral nutrients (Ca, Zn and Fe) were observed in the field (Table 3, Fig. 3). Given that LRD and field growth traits were not negatively associated, it is likely that varieties with greater LRD were able to acquire these nutrients more efficiently. This hypothesis could now be tested directly.

Roots of nutrient-limited plants growing through soils with heterogeneous nutrient availability often show increased LRD in places with greater nutrient supply (Drew, 1975; Robinson, 1994; Lopez-Bucio *et al.*, 2003; Hodge, 2004; Ostonen *et al.*, 2007). Whilst varieties of OSR in the current study will undoubtedly respond to soil nutrient heterogeneity, it seems unlikely that plants were nutrient-deficient on these well-fertilized soils, where the mineral composition of leaves was in a range defined as sufficient for plant growth (White and Brown, 2010). Thus, given that it is not possible to measure fine lateral roots of OSR in the field, the LRD trait warrants further study in HTP systems, with the following foci: (1) determining the effects of LRD on nutrient uptake efficiencies under resource-limited and resource-adequate conditions; and (2) quantifying wider sources of genetic variation in LRD in adapted and non-adapted OSR material to test for selective value in breeding. The inverse relationship between LRD and MLRL in the HTP system is also worth further exploration in terms of trade-offs between resource allocation and acquisition. Adaptive responses to low nutrient availability could also include increased root hair formation, secretion of lytic enzymes, and the release of organic acids and other metabolites into the rhizosphere (Osmont *et al*., 2007; Gruber *et al.*, 2013; White *et al.*, 2013*a*).

Most variation in traits measured in the HTP system was due to residual factors (plant-to-plant variation) highlighting the variability of these root traits, the need to control environmental factors and the need for high replication. This is consistent with the observations of Adu *et al*. (2014), who reported seedling root trait phenotypes of 16 *B. rapa* genotypes grown on germination paper and imaged using high-resolution scanners. The data presented here indicate that between 11 and 36 replicates of OSR would be needed to detect a 30 % difference in root traits in both HTP systems and field environments (Fig. 4). Adu *et al*. (2014) reported that between 6 and 43 replicates would be needed to detect a 50 % difference in root traits grown in their HTP system. In both these studies, the number of replicates required increased in the order PRL < LRD < TRL < MLRL, with varietal factors explaining most variation in PRL and least variation in MLRL. Thus, PRL is the most reliable trait to detect genotypic differences in the HTP system and MLRL the least reliable. It seems likely that residual variation will decrease when wider panels of varieties are used, whilst some of the residual variation will be due to measurable factors such as variation in seed size at the individual plant-to-plant scale (Evans and Bhatt, 1977; Khan *et al*., 2012). Both of these hypotheses are currently being tested in ongoing work.

## SUPPLEMENTARY DATA

Supplementary data are available online at www.aob.oxfordjournals.org and consist of the following. Table S1: all root traits of *Brassica napus* grown in the HTP system, and yield, emergence and root and shoot traits in field experiments. Table S2: leaf mineral concentration of *Brassica napus* grown in the field at Bingham (2013–14).

Supplementary Data
