# Supplementary material for: High-throughput phenotyping (HTP) identifies seedling root traits linked to variation in seed yield and nutrient capture in field-grown oilseed rape (Brassica napus L.)
Source: Ann Bot. 2016 Apr 6;118(4):655–65. doi: 10.1093/aob/mcw046 (PMC5055618; doi:10.1093/aob/mcw046)
Supplement: Supplementary Data [file supp_118_4_655__index.html]

High-throughput phenotyping (HTP) identifies seedling root traits linked to variation in seed yield and nutrient capture in field-grown oilseed rape (Brassica napus L.) — Supplementary Data 

# High-throughput phenotyping (HTP) identifies seedling root traits linked to variation in seed yield and nutrient capture in field-grown oilseed rape (*Brassica napus* L.)

## Supplementary Data

files

- Supplementary Data - xlsx file
